# Supplementary material for: Codon-specific Ramachandran plots show amino acid backbone conformation depends on identity of the translated codon
Source: Nat Commun. 2022 May 20;13:2815. doi: 10.1038/s41467-022-30390-9 (PMC9123026; doi:10.1038/s41467-022-30390-9)
Supplement: Supplementary file 1 — Supplementary Information [file 41467_2022_30390_MOESM1_ESM.pdf]

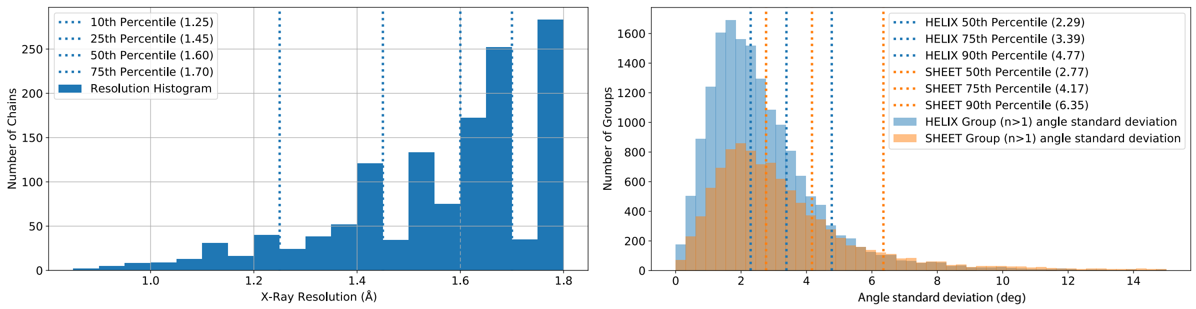


**Supplementary Figure 1 – Histograms of data collection.** Left: distribution of structure resolutions. Right: distribution of dihedral angle standard deviations for groups of unique Uniprot ID:location with more than one PDB structure available. The standard deviation is measured on the $\varphi,\psi$ torus.

| Codon | HELIX | SHEET |
|---|---|---|
| A-GCA | 1258 | 390 |
| A-GCC | 1607 | 529 |
| A-GCG | 2157 | 658 |
| A-GCT | 994 | 333 |
| C-TGC | 193 | 191 |
| C-TGT | 167 | 148 |
| D-GAC | 805 | 328 |
| D-GAT | 1347 | 564 |
| E-GAA | 2374 | 814 |
| E-GAG | 1161 | 343 |
| F-TTC | 677 | 571 |
| F-TTT | 888 | 797 |
| G-GGA | 153 | 163 |
| G-GGC | 549 | 578 |
| G-GGG | 245 | 194 |
| G-GGT | 462 | 547 |
| H-CAC | 378 | 265 |
| H-CAT | 510 | 354 |
| I-ATA | 185 | 168 |
| I-ATC | 1088 | 1093 |
| I-ATT | 1290 | 1421 |
| K-AAA | 1790 | 787 |
| K-AAG | 509 | 243 |
| L-CTA | 169 | 94 |
| L-CTC | 528 | 296 |
| L-CTG | 2812 | 1478 |
| L-CTT | 530 | 297 |
| L-TTA | 641 | 332 |
| L-TTG | 599 | 395 |
| M-ATG | 1187 | 572 |

| Codon | HELIX | SHEET |
|---|---|---|
| N-AAC | 782 | 411 |
| N-AAT | 623 | 311 |
| P-CCA | 184 | 123 |
| P-CCC | 92 | 61 |
| P-CCG | 492 | 294 |
| P-CCT | 151 | 90 |
| Q-CAA | 828 | 283 |
| Q-CAG | 1629 | 574 |
| R-AGA | 103 | 48 |
| R-AGG | 63 | 27 |
| R-CGA | 140 | 81 |
| R-CGC | 1066 | 490 |
| R-CGG | 222 | 111 |
| R-CGT | 1039 | 448 |
| S-AGC | 503 | 313 |
| S-AGT | 264 | 186 |
| S-TCA | 216 | 145 |
| S-TCC | 301 | 211 |
| S-TCG | 261 | 166 |
| S-TCT | 302 | 223 |
| T-ACA | 215 | 243 |
| T-ACC | 846 | 870 |
| T-ACG | 461 | 400 |
| T-ACT | 334 | 337 |
| V-GTA | 474 | 547 |
| V-GTC | 534 | 758 |
| V-GTG | 986 | 1369 |
| V-GTT | 733 | 1049 |
| W-TGG | 660 | 534 |
| Y-TAC | 586 | 501 |
| Y-TAT | 667 | 657 |

**Supplementary Table 1 – Sample sizes** of each codon group in the $\alpha$-helix and β-sheet secondary structures.

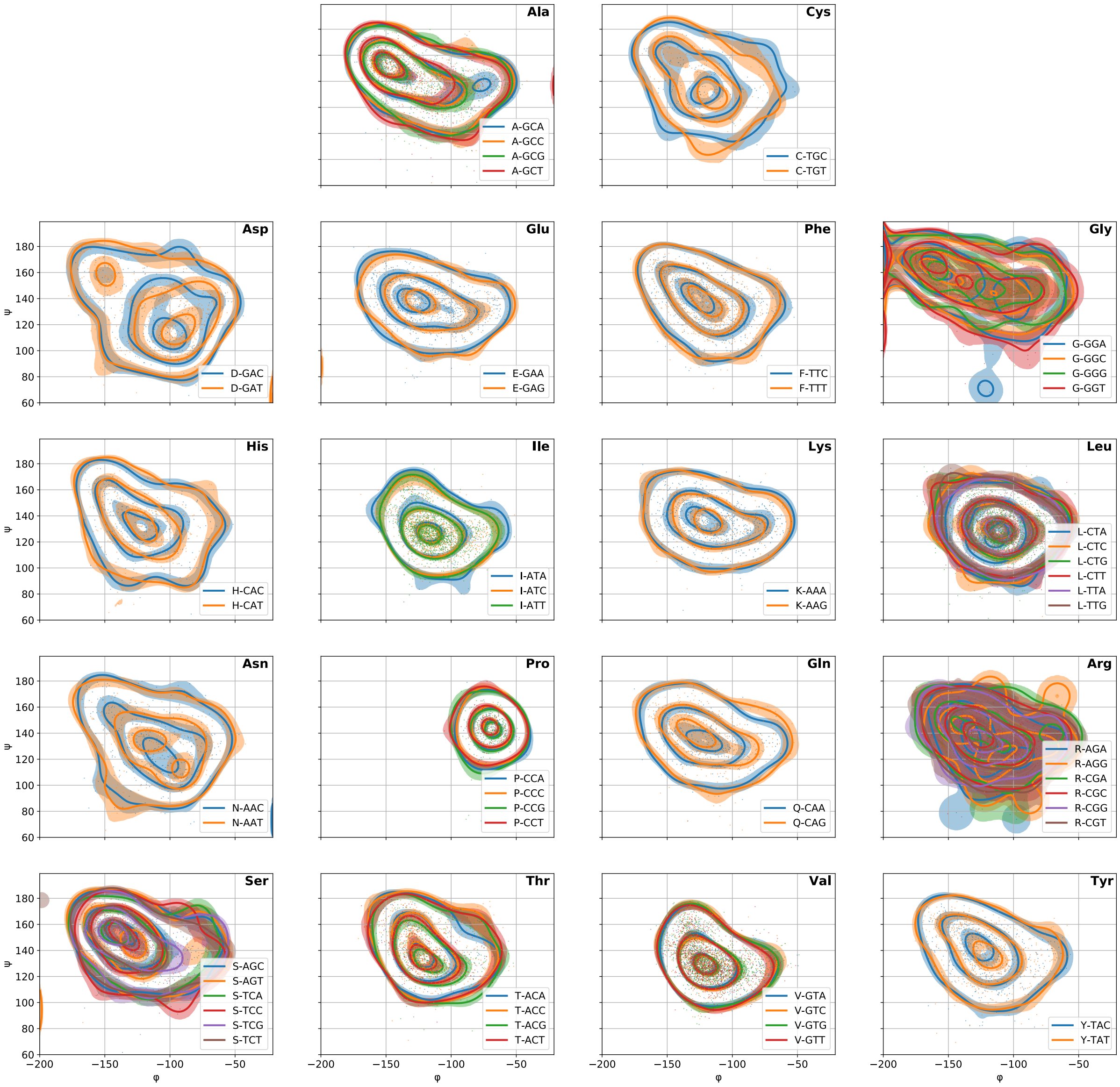


**Supplementary Figure 2 – Codon-specific Ramachandran plots of 18 amino acids encoded by more than one codon in the β-mode.** Contour plots depict the level lines containing 10%, 50% and 90% of the probability mass. Shaded regions represent 10%-90% confidence intervals calculated on 1000 random bootstraps.

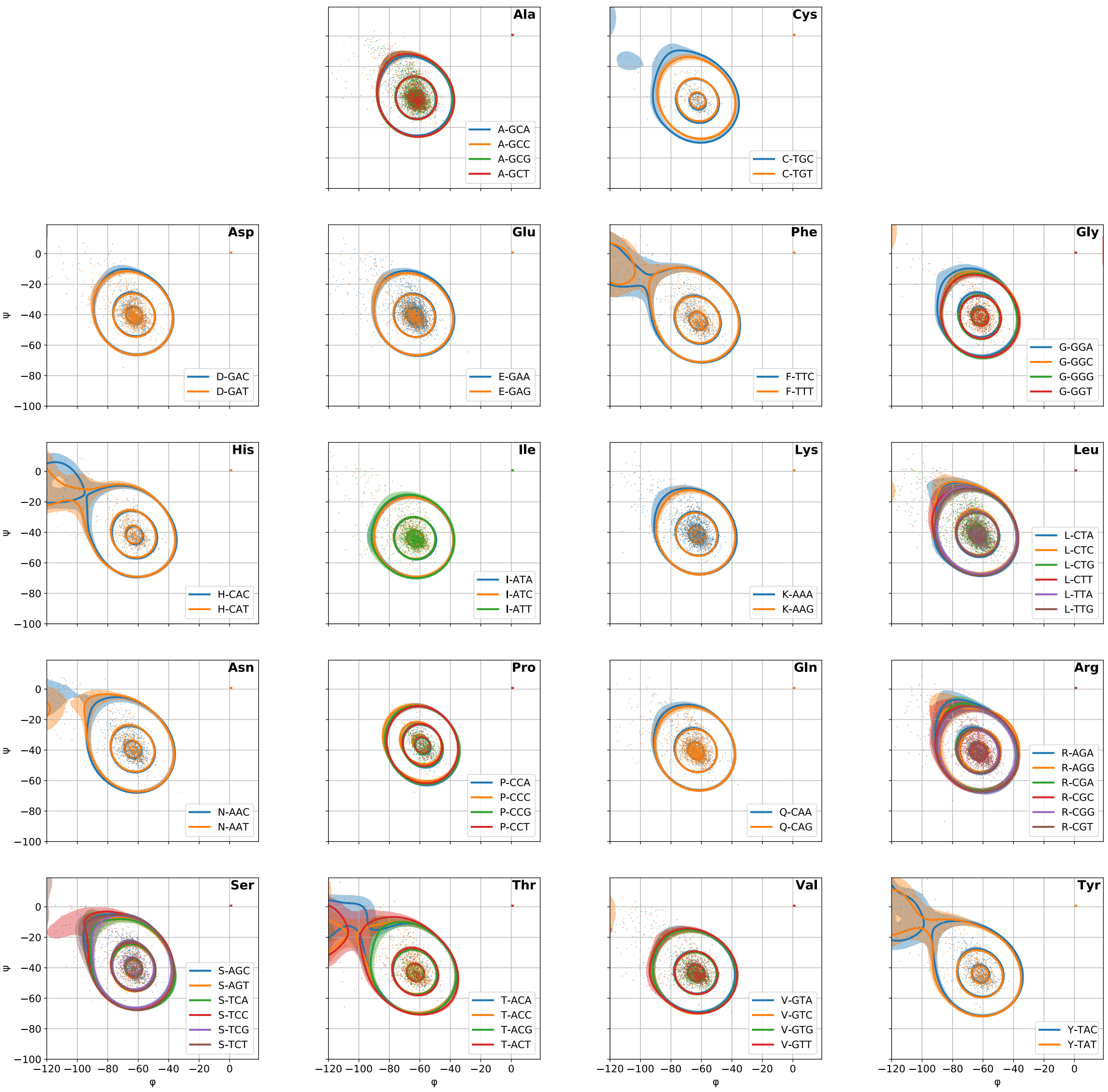


**Supplementary Figure 3 – Codon-specific Ramachandran plots of 18 amino acids encoded by more than one codon in the α-mode.** Contour plots depict the level lines containing 10%, 50% and 90% of the probability mass. Shaded regions represent 10%-90% confidence intervals calculated on 1000 random bootstraps.

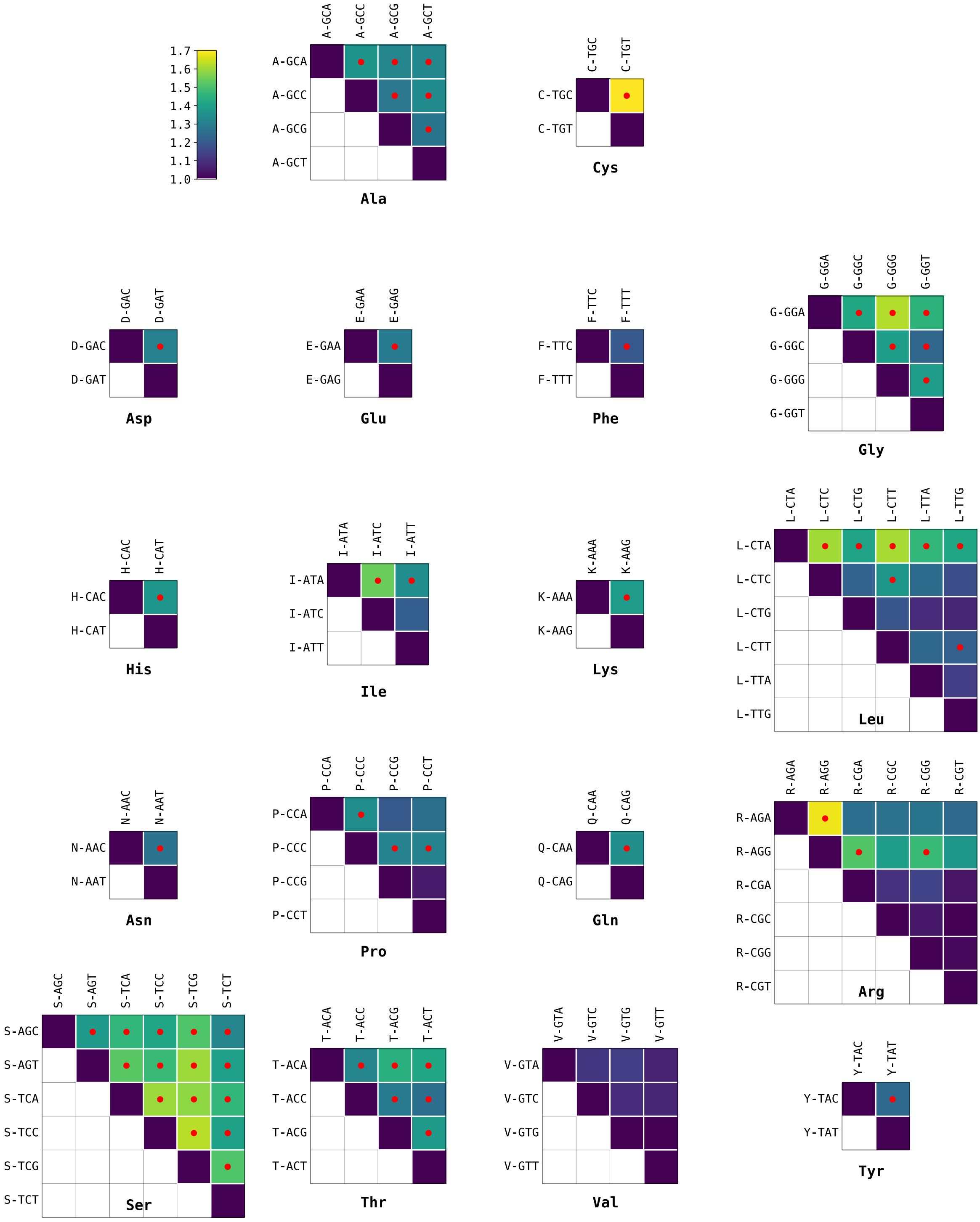


**Supplementary Figure 4 – Normalized pair-wise distances between codon-specific angle distributions in the β-mode.** Pairs for which the null hypothesis was rejected are marked with a red dot.

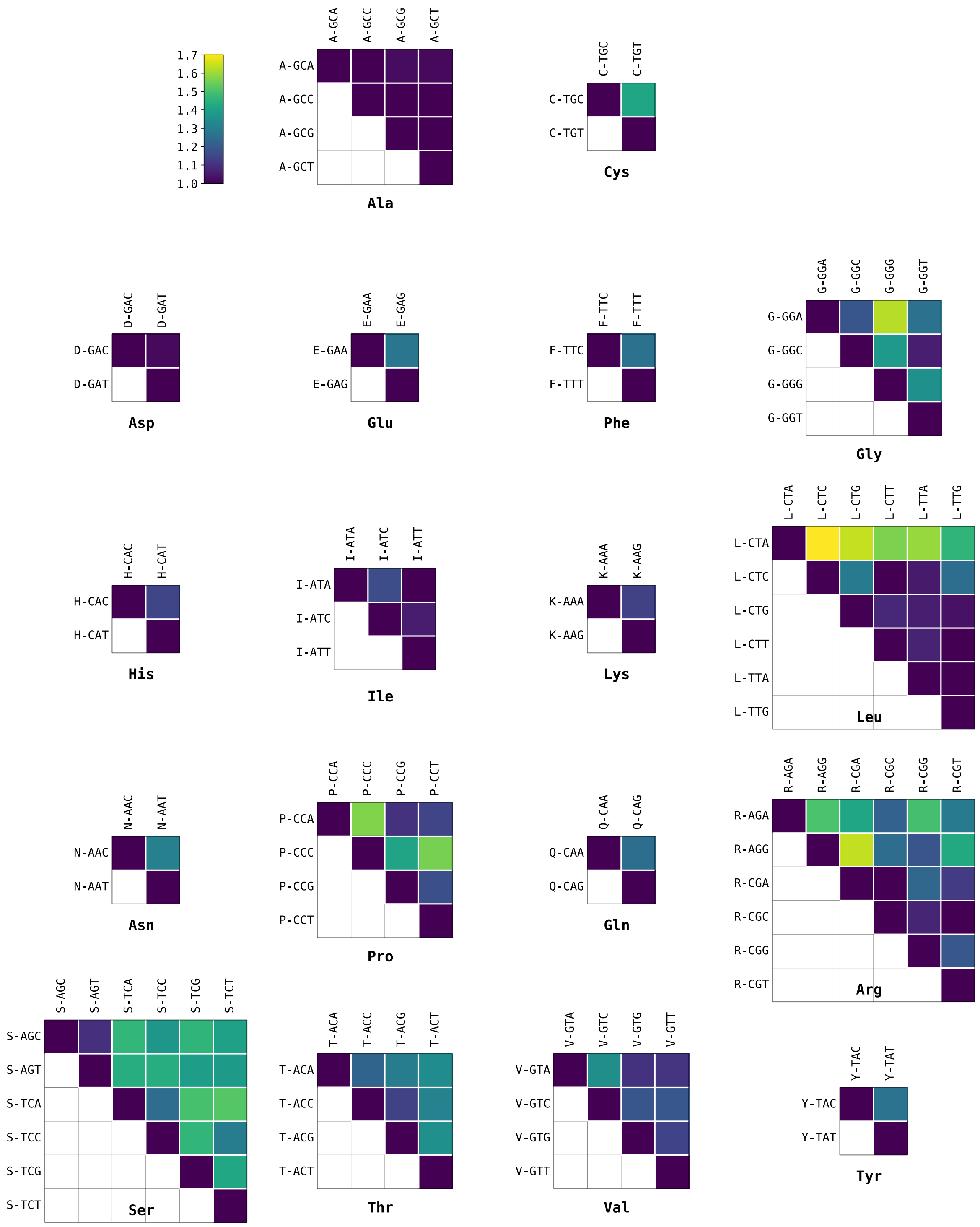


**Supplementary Figure 5 – Normalized pair-wise distances between codon-specific angle distributions in the $\alpha$-mode.** The null hypothesis was rejected on no pairs.

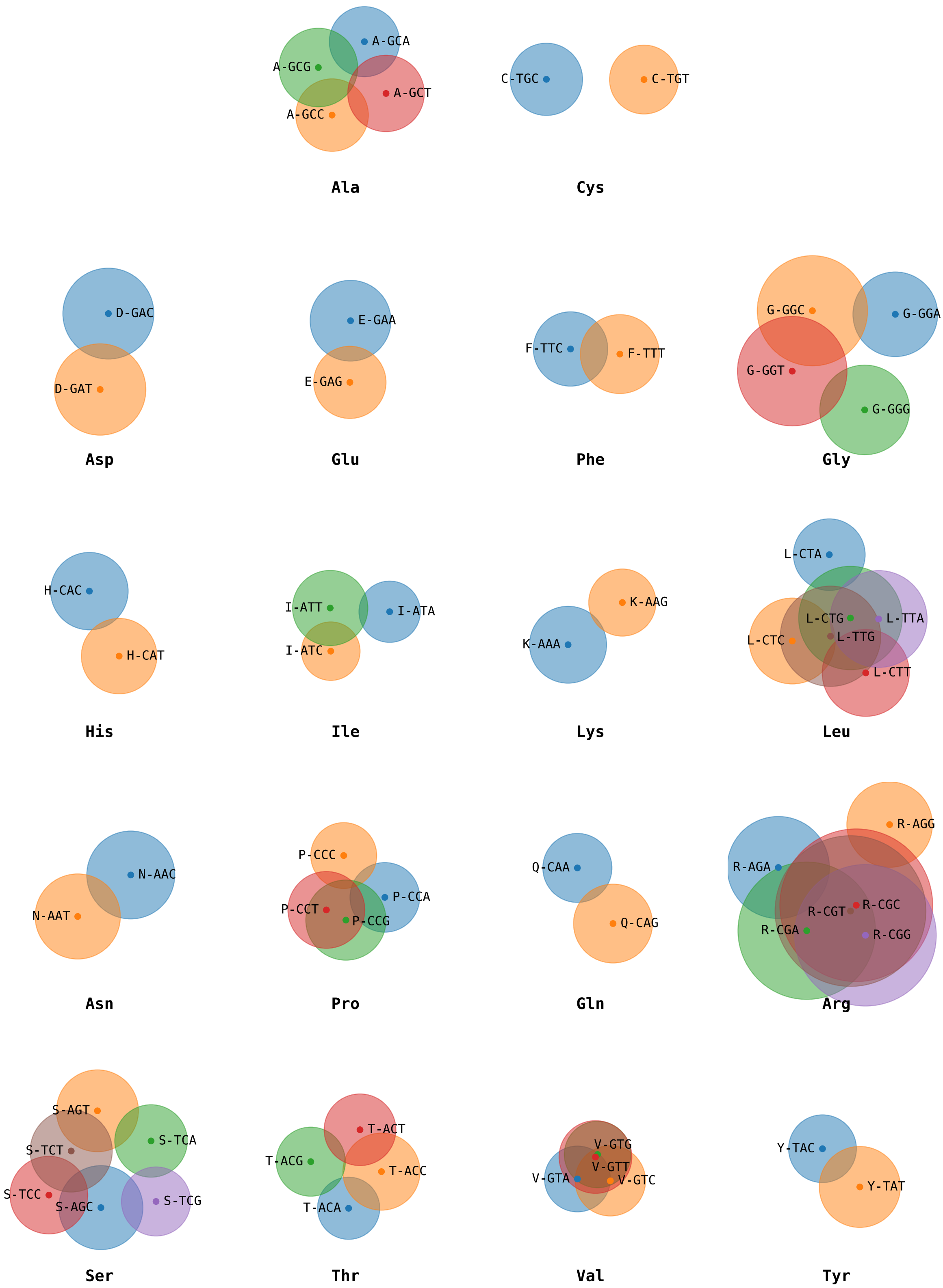


**Supplementary Figure 6 – MDS plots approximating pair-wise distances between codon-specific angle distributions in the β-mode.**

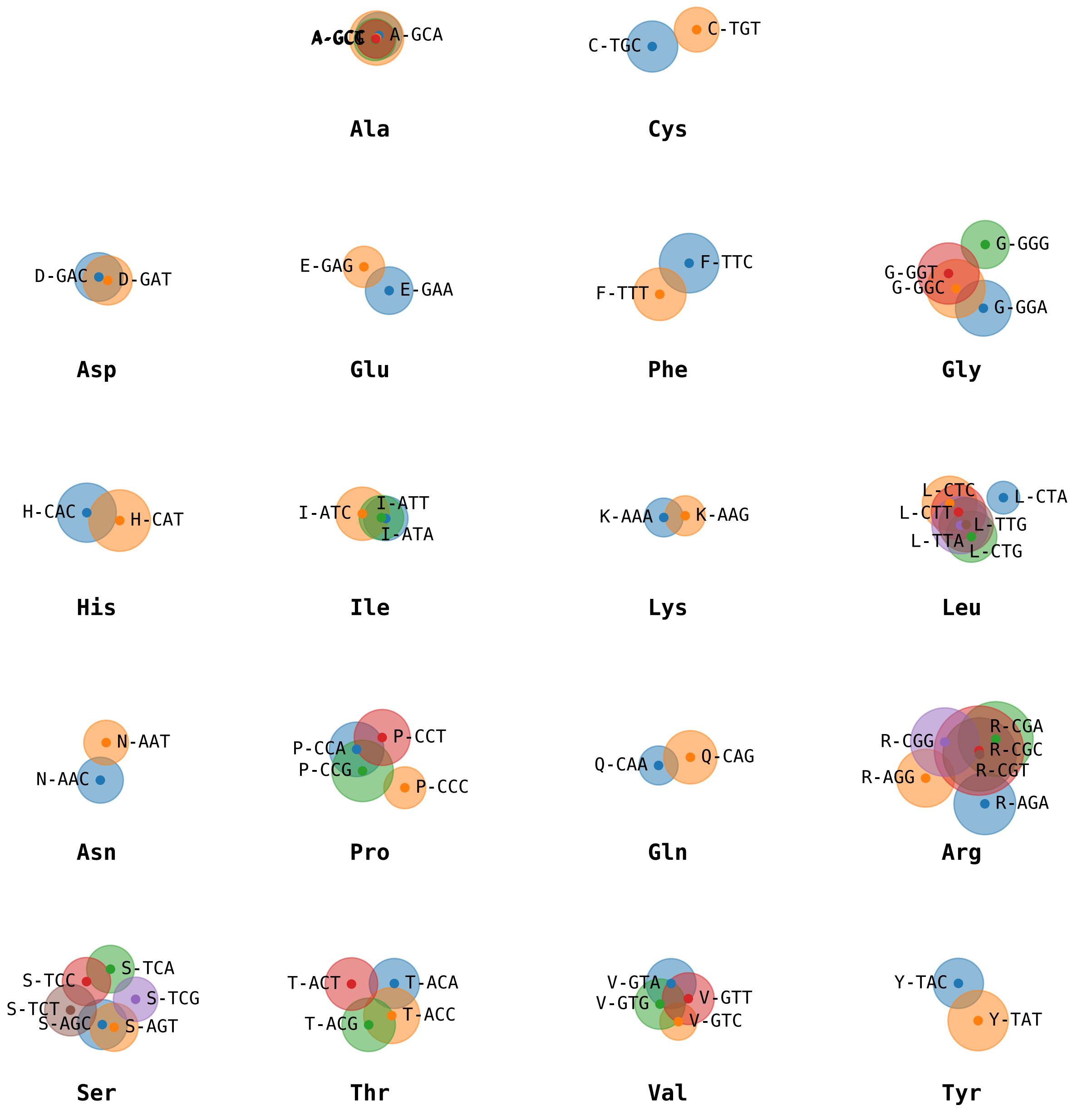


**Supplementary Figure 7 – MDS plots approximating pair-wise distances between codon-specific angle distributions in the $\alpha$-mode.**

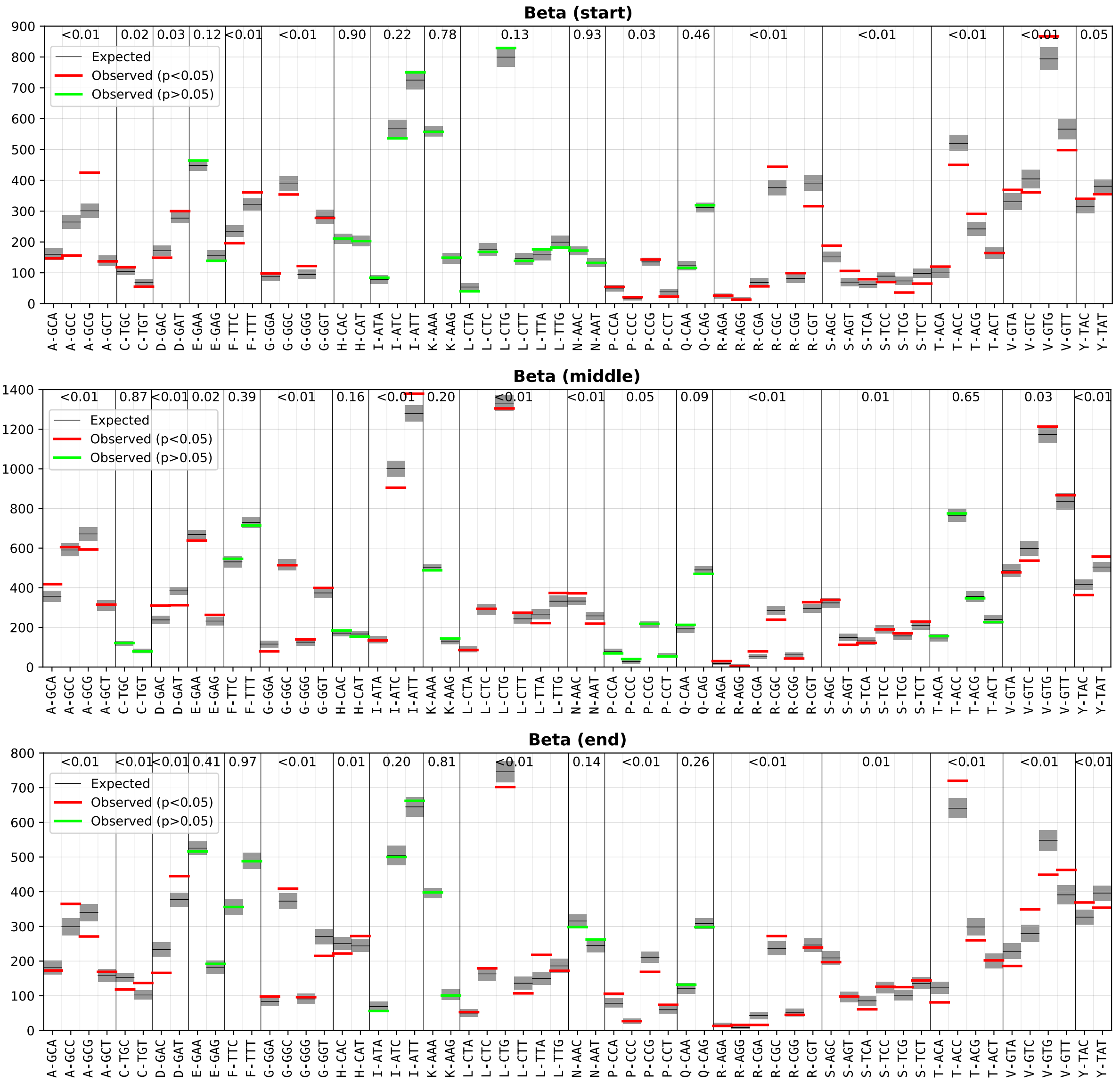


**Supplementary Figure 8 – Expected and observed number of codons in three secondary sub-structures of the β-mode (first two, last two and all middle residues of β-strands).** Red lines indicate codons for which the abundance in the sub-β-mode is significantly different ($p<0.05$) from the abundance observed in the full β-mode. The confidence intervals on the expected number of occurrences represent 5%- and 95%-tiles. For significance evaluation, p-values (displayed on the top) were calculated using the two-sided G-test per amino acid in every sub-structure.

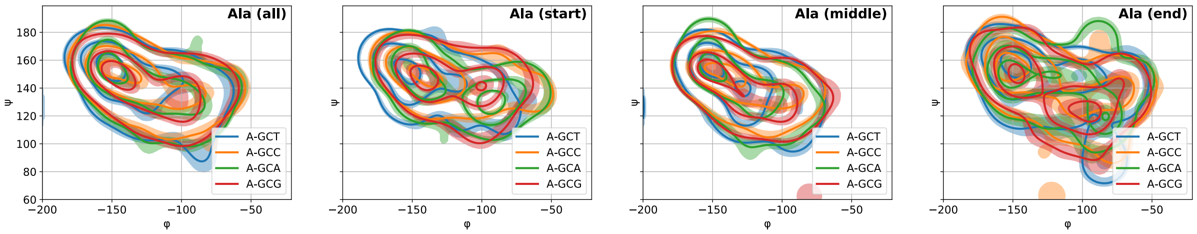


**Supplementary Figure 9 – Synonymous codon distributions of an abundant amino acid (alanine) in the three secondary sub-structures of the β-mode (first two, last two and all middle residues of a β-sheet).** This amino acid was selected due to its large sample size and because it exhibits significantly different codon abundance in the β-mode sub-structures compared to its expected abundance in the full β-mode (see Supplementary Fig. 8). Dihedral angle distribution differences are still observed between synonymous codons, even after conditioning by finer secondary sub-structure. Contour plots depict the level lines containing 10%, 50% and 90% of the probability mass. Shaded regions represent 10%-90% confidence intervals calculated on 1000 random bootstraps.
