# Supplementary material for: Codon-specific Ramachandran plots show amino acid backbone conformation depends on identity of the translated codon
Source: Nat Commun. 2022 May 20;13:2815. doi: 10.1038/s41467-022-30390-9 (PMC9123026; doi:10.1038/s41467-022-30390-9)
Supplement: Supplementary file 3 — Reporting Summary [file 41467_2022_30390_MOESM3_ESM.pdf]

nature portfolio

Corresponding author(s): Alexander Bronstein

Last updated by author(s): Mar 10, 2022

Reporting Summary

Nature Portfolio wishes to improve the reproducibility of the work that we publish. This form provides structure for consistency and transparency in reporting. For further information on Nature Portfolio policies, see our Editorial Policies and the Editorial Policy Checklist.

Statistics

For all statistical analyses, confirm that the following items are present in the figure legend, table legend, main text, or Methods section.

n/a	Confirmed	
☐	☒	The exact sample size (n) for each experimental group/condition, given as a discrete number and unit of measurement
☐	☒	A statement on whether measurements were taken from distinct samples or whether the same sample was measured repeatedly
☐	☒	The statistical test(s) used AND whether they are one- or two-sided<br>*Only common tests should be described solely by name; describe more complex techniques in the Methods section.*
☐	☒	A description of all covariates tested
☐	☒	A description of any assumptions or corrections, such as tests of normality and adjustment for multiple comparisons
☐	☒	A full description of the statistical parameters including central tendency (e.g. means) or other basic estimates (e.g. regression coefficient) AND variation (e.g. standard deviation) or associated estimates of uncertainty (e.g. confidence intervals)
☐	☒	For null hypothesis testing, the test statistic (e.g. F, t, r) with confidence intervals, effect sizes, degrees of freedom and P value noted<br>*Give P values as exact values whenever suitable.*
☒	☐	For Bayesian analysis, information on the choice of priors and Markov chain Monte Carlo settings
☒	☐	For hierarchical and complex designs, identification of the appropriate level for tests and full reporting of outcomes
☐	☒	Estimates of effect sizes (e.g. Cohen's d, Pearson's r), indicating how they were calculated

Our web collection on statistics for biologists contains articles on many of the points above.

Software and code

Policy information about availability of computer code

Data collection	The code implementing the described data collection and analysis methods has been deposited in the XXZenodo repository [https://doi.org/10.5281/zenodo.6345285link]. Code is available under restricted access conditioned on user identification and agreement to academic use license. To access the repository, please use the provided DOI URL.
Data analysis	Custom code was used based on open source python packages. The full code will be made available upon publication.<br>Main software package versions (full environment specification is supplied with the code):<br>python 3.8.12<br>biopython 1.79<br>blast 2.12<br>dssp 3.0.0<br>numpy 1.21.5<br>pandas 1.4.1<br>matplotlib 3.5.1

For manuscripts utilizing custom algorithms or software that are central to the research but not yet described in published literature, software must be made available to editors and reviewers. We strongly encourage code deposition in a community repository (e.g. GitHub). See the Nature Portfolio guidelines for submitting code & software for further information.

Data

Policy information about availability of data

All manuscripts must include a data availability statement. This statement should provide the following information, where applicable:

- Accession codes, unique identifiers, or web links for publicly available datasets
- A description of any restrictions on data availability
- For clinical datasets or third party data, please ensure that the statement adheres to our policy

The protein records data collected for this study as well as full output datasets have been deposited in the Harvard Dataverse database [https://doi.org/10.7910/DVN/5P81D4].

Field-specific reporting

Please select the one below that is the best fit for your research. If you are not sure, read the appropriate sections before making your selection.

[x] Life sciences [ ] Behavioural & social sciences [ ] Ecological, evolutionary & environmental sciences

For a reference copy of the document with all sections, see nature.com/documents/nr-reporting-summary-flat.pdf

Life sciences study design

All studies must disclose on these points even when the disclosure is negative.

Sample size	Sample sizes were determined by the maximal amount of PDB data with sufficient resolution and unambiguous codon and DSSP assignment.
Data exclusions	Data exclusion criteria were predetermined and rationalized. PDB structures were filtered by source organism (to facilitate assigning an accurate sequence), resolution (to allow enough accuracy in atomic coordinates), homology (to avoid over-representation of particular structure types) and secondary structure (to factor out the known phenomenon that codons are over or under represented in particular secondary structures).
Replication	We are going to publish our code and are providing the full dataset that produced the figures in the paper. All reported results are produced by this code.
Randomization	We used randomization for bootstrap sampling and permutation testing with repeatable seeds. Full information about the procedures is reported.
Blinding	Blinding is not relevant in our studies. For the data analysis we predetermined objective criteria (based on acceptable standards in the field or custom calibrated for the purpose. Subsequently, large scale data collection and analysis was carried out automatically.

Reporting for specific materials, systems and methods

We require information from authors about some types of materials, experimental systems and methods used in many studies. Here, indicate whether each material, system or method listed is relevant to your study. If you are not sure if a list item applies to your research, read the appropriate section before selecting a response.

Materials & experimental systems

n/a	Involved in the study	
[x]	[ ]	Antibodies
[x]	[ ]	Eukaryotic cell lines
[x]	[ ]	Palaeontology and archaeology
[x]	[ ]	Animals and other organisms
[x]	[ ]	Human research participants
[x]	[ ]	Clinical data
[x]	[ ]	Dual use research of concern

Methods

n/a	Involved in the study	
[x]	[ ]	ChIP-seq
[x]	[ ]	Flow cytometry
[x]	[ ]	MRI-based neuroimaging
